# Data on IL-10R neutralization-induced chronic colitis in Lipocalin 2 deficient mice on BALB/c background

**DOI:** 10.1016/j.dib.2017.03.002

**Published:** 2017-03-08

**Authors:** Vishal Singh, Beng San Yeoh, Marit Nilsen-Hamilton, Thorsten Berger, Tak W. Mak, Matam Vijay-Kumar

**Affiliations:** a Department of Nutritional Sciences, The Pennsylvania State University, University Park, PA 16802, USA; b Department of Biochemistry, Biophysics and Molecular Biology, Iowa State University, Ames, IA 50011, USA; c The Campbell Family Institute for Breast Cancer Research, University Health Network, Toronto, ON, Canada M5G 2C1

**Keywords:** Siderocalin, Neutrophil gelatinase-associated lipocalin, Inflammatory bowel disease, IL-10

## Abstract

The data herein is related to the research article entitled “Microbiota-inducible Innate Immune, Siderophore Binding Protein Lipocalin 2 is Critical for Intestinal Homeostasis” (Singh et al., 2016) [1] where we have demonstrated that C57BL/6 Lipocalin 2 deficient mice (*Lcn2*KO) developed chronic colitis upon anti-interleukin-10 receptor (αIL-10R) monoclonal antibody administration. In the present article, we evaluated the susceptibility of BALB/c *Lcn2*KO mice and their WT littermates to the αIL-10R neutralization-induced chronic colitis. Our data showed that αIL-10R mAb-treated BALB/c *Lcn2*KO mice exhibited severe chronic colitis (i.e., splenomegaly, colomegaly, colonic pathology, and incidence of rectal prolapse) when compared to WT mice.

**Specifications Table**

**Table:** 

Subject area	*Biology*
More specific subject area	*Lipocalin 2, inflammatory bowel disease*
Type of data	*Graphs, images, figures*
How data was acquired	*Assessment of colitis parameters: splenomegaly, colomegaly, colon histology, enzyme-linked immunosorbent assay (ELISA), and quantitative reverse-transcription polymerase chain reaction (qRT-PCR). Biotek Eon*^*™*^*microplate spectrophotometer and Step One Plus Real-Time PCR System.*
Data format	*Analyzed*
Experimental factors	*Lcn2KO mice and their WT littermates were treated with anti-IL-10R monoclonal antibody or anti-IgG isotype control as described in Ref*. [1]
Experimental features	*Analysis of standard colitis parameters*
Data source location	*Pennsylvania, USA*
Data accessibility	*Data are provided with this article*

**Value of the data**

• The data are valuable to researchers interested in investigating the role of lipocalin 2 (Lcn2) in inflammatory bowel disease.
• The data indicate that Lcn2 deficiency predisposes to colitis and this phenotype can be recapitulated in mice on the BALB/c background.
• The data support future studies in delineating the role of Lcn2 in conferring mucoprotection.

## 1 Data

**Fig. 1:**
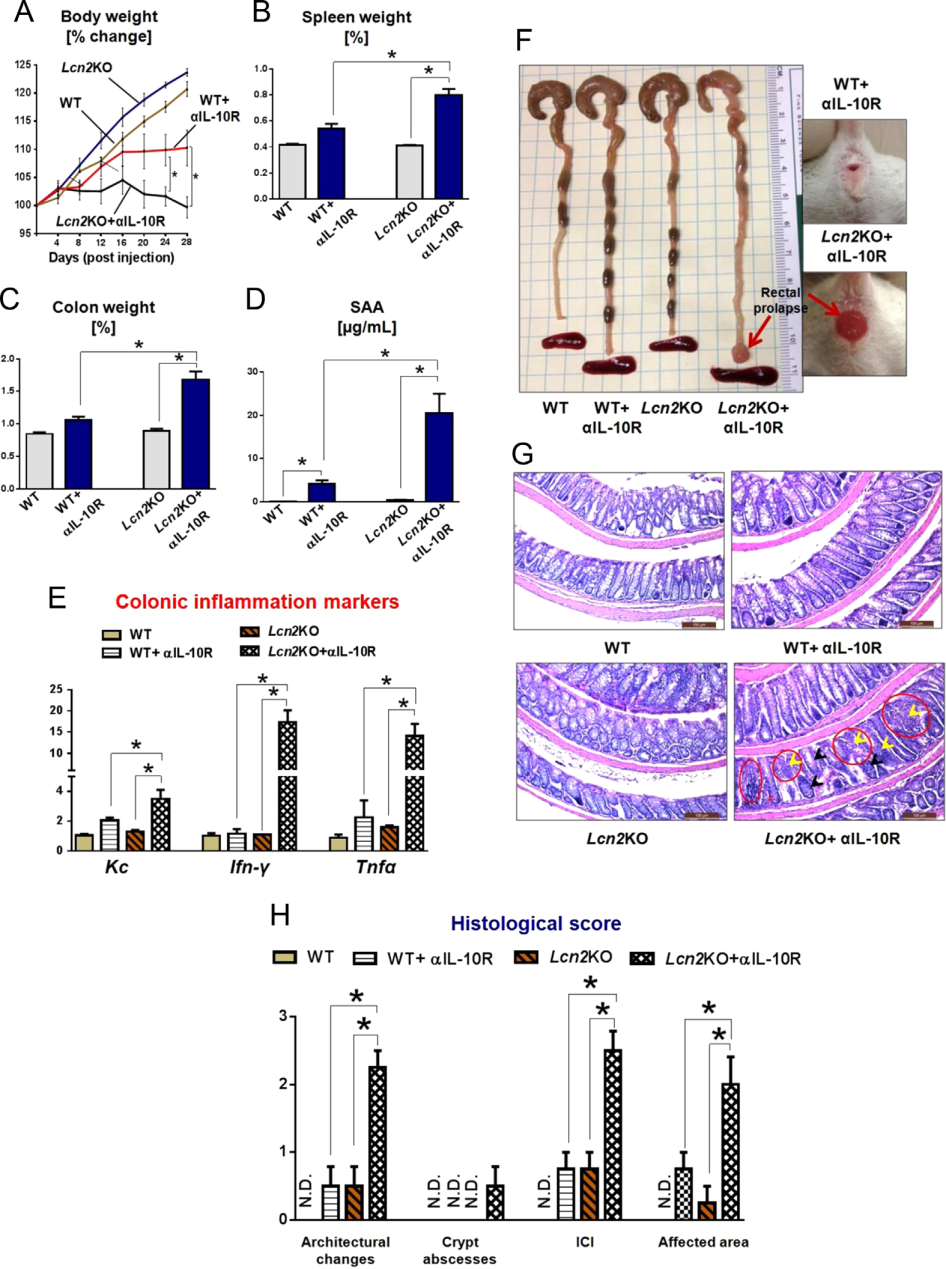
Lcn2 deficiency aggravates colitis upon inhibition of IL-10R signaling. Lcn2-deficient mice (*Lcn2*KO) and WT littermates (male, *n*=4) on BALB/c background were weaned on day 21, and at four weeks of age mice were given interleukin-10 receptor (IL-10R) neutralizing antibody (1.0 mg/mouse, 4 weekly injections; i.p., BioXcell) and monitored for body weights regularly. Control mice received isotype control antibody (rat anti-mouse IgG1). One week post last injection, the mice were euthanized and analyzed for colitis parameters. (A) Line graph represents percent change in body weight. (B and C) Bar graphs represent percent (B) spleen weight and (C) colon weight. (D) Graph display circulating level of serum amyloid A (SAA), a marker of active inflammation, measured by ELISA. Colons were harvested, emptied of fecal contents, opened longitudinally and washed in ice-cold PBS. A portion of the proximal colon was collected for qPCR analysis, the remaining colon was used to make Swiss roll for histological analysis. (E) Relative mRNA level of pro-inflammatory cytokines in the colon. (F) Representative colon images from control and αIL-10R treated WT and *Lcn2*KO mice. Right-side panel shows the rectal prolapse (red arrow) in *Lcn2*KO mice. (G) Image display hematoxylin and eosin (H&E)-stained colonic sections. Red circle shows the inflammatory cell infiltrate (ICI) in the lamina propria and crypt architectural distortion. Black and yellow arrowhead show goblet cell loss and crypt loss respectively. (H) Histological score was assessed by visualizing the entire H&E-stained colon sections microscopically for the extent of ICI in lamina propria, mucosa and submucosa, epithelial hyperplasia, goblet cell loss, distorted crypt structure, ulcerations and crypt loss. The values are expressed as mean±SEM (*p<0.05).

The dataset of this article provides additional information to Ref. [1], in which we have characterized the increased susceptibility of C57BL/6 lipocalin 2 knockout (*Lcn2*KO) mice to colitis. Considering that the mouse genetic background can influence colitogenesis [i.e., immune responses of C57BL/6 and BALB/c mice are Th1 and Th2-biased, respectively [2], [3], [4], [5], we herein investigated the susceptibility of BALB/c *Lcn2*KO mice to interleukin-10 receptor (IL-10R) neutralization-induced chronic colitis. The data presented here elucidate that the robust chronic colitis observed in αIL-10R-treated C57BL/6 *Lcn2*KO mice [1] can be recapitulated in BALB/c *Lcn2*KO mice. Specifically, splenomegaly, colomegaly, and elevated serum and colonic inflammation markers were observed in αIL-10R-treated BALB/c *Lcn2*KO mice when compared to their respective WT control (Fig. 1A–E). Remarkably, BALB/c *Lcn2*KO mice exhibited rectal prolapse, a severe form of colitis, upon IL-10R neutralization (Fig. 1F). Histological analysis—extent of inflammatory cell infiltrate (ICI), epithelial hyperplasia, goblet cell loss, and distorted crypt structure (Fig. 1G and H)— further established that BALB/c *Lcn2*KO mice develop a severe chronic colitis, upon IL-10R neutralization, when compared to WT control.

## 2 Experimental design, materials and methods

### 2.1 Mice

*Lcn2*KO mice [6] and their WT littermates on BALB/c background were maintained under specific-pathogen-free conditions in the animal house facility at Pennsylvania State University, PA. Mice were housed in cages (max. 5 mice per cage) and fed on chow-control diet *ad libitum* with unrestricted access to water. Animal experiments were approved by the Institutional Animal Care and Use Committee (IACUC) of Pennsylvania State University. Gut microbiota composition was analyzed in BALB/c Lcn2KO mice and their WT littermates as described in ref. [1].

### 2.2 IL-10R neutralization-induced chronic colitis

Four weeks old BALB/c *Lcn2*KO mice and their WT littermates (*n*=4) were administered with four weekly injections (1.0 mg/mouse, intraperitoneally) of anti-mouse αIL-10R mAb (BioXcell). Control mice were administered with the isotype (IgG1) control antibody. Colonic inflammation was examined by monitoring for body weight, fecal occult blood, and diarrhea. At one week after the last injection of αIL-10R mAb or IgG1 control, the mice were euthanized by CO_2_ asphyxiation and assessed for standard chronic colitis parameters.

### 2.3 Enzyme-linked immunosorbent assay

Blood samples were collected in a BD Microtainer (Becton Dickinson) via the retro-orbital plexus at euthanasia. Hemolysis-free sera were collected after centrifugation and stored at −80 °C until further analysis. Serum amyloid A (SAA) level was analyzed by ELISA according to the manufacturer׳s (R & D Systems) protocol.

### 2.4 Quantitative reverse-transcription PCR

Total RNA was isolated from colonic tissue using TRI reagent (Sigma) and used to synthesize cDNA using the cDNA Synthesis Kit (Quanta BioSciences). qRT-PCR was performed with the use of SYBR Green Master Mix (Quanta Bio-Sciences) and primers specific for mouse *TNF* and *36B4* as described in Ref. [1], and read using the Step One Plus Real-Time PCR Q28 System (Applied Biosystems).

### 2.5 Histology

After euthanasia, mouse colons were prepared as Swiss roll, fixed overnight in 10% neutral buffered formalin and stored in 70% ethanol. Colons were processed for paraffin embedding and serial sections (5 mm) were collected and stained with hematoxylin and eosin (H&E) at the Animal Diagnostic Laboratory, PSU. Histologic scoring was performed as described previously [7].

### 2.6 Statistical analysis

Data are presented as means±SEM. Statistical significance between the groups was calculated using a one-way ANOVA followed by Tukey׳s multiple comparison test. *P*<0.05 was considered statistically significant. All statistical analyses were performed with the GraphPad Prism 7.0 program (GraphPad, Inc.).

## References

[1] Singh V., Yeoh B.S., Chassaing B., Zhang B., Saha P., Xiao X., Awasthi D., Shashidharamurthy R., Dikshit M., Gewirtz A., Vijay-Kumar M. (2016). Microbiota-inducible innate immune, Siderophore Binding Protein Lipocalin 2 is critical for intestinal homeostasis. Cell. Mol. Gastroenterol. Hepatol..

[2] Gorham J.D., Guler M.L., Steen R.G., Mackey A.J., Daly M.J., Frederick K., Dietrich W.F., Murphy K.M. (1996). Genetic mapping of a murine locus controlling development of T helper 1/T helper 2 type responses. Proc. Natl. Acad. Sci. USA.

[3] Yagi J., Arimura Y., Takatori H., Nakajima H., Iwamoto I., Uchiyama T. (2006). Genetic background influences Th cell differentiation by controlling the capacity for IL-2-induced IL-4 production by naive CD4+ T cells. Int. Immunol..

[4] Trunova G.V., Makarova O.V., Diatroptov M.E., Bogdanova I.M., Mikchailova L.P., Abdulaeva S.O. (2011). Morphofunctional characteristic of the immune system in BALB/c and C57BL/6 mice. Bull. Exp. Biol. Med..

[5] Chen X., Oppenheim J.J., Howard O.M. (2005). BALB/c mice have more CD4+CD25+ T regulatory cells and show greater susceptibility to suppression of their CD4+CD25- responder T cells than C57BL/6 mice. J. Leukoc. Biol..

[6] Berger T., Togawa A., Duncan G.S., Elia A.J., You-Ten A., Wakeham A., Fong H.E., Cheung C.C., Mak T.W. (2006). Lipocalin 2-deficient mice exhibit increased sensitivity to Escherichia coli infection but not to ischemia-reperfusion injury. Proc. Natl. Acad. Sci. USA.

[7] Erben U., Loddenkemper C., Doerfel K., Spieckermann S., Haller D., Heimesaat M.M., Zeitz M., Siegmund B., Kuhl A.A. (2014). A guide to histomorphological evaluation of intestinal inflammation in mouse models. Int. J. Clin. Exp. Pathol..

