# Supplementary material for: Data on IL-10R neutralization-induced chronic colitis in Lipocalin 2 deficient mice on BALB/c background
Source: Data Brief. 2017 Mar 8;11:588–92. doi: 10.1016/j.dib.2017.03.002 (PMC5358532; doi:10.1016/j.dib.2017.03.002)
Supplement: Supplementary file 2 — Supplementary material [file mmc2.docx]

**Supplementary data**

**
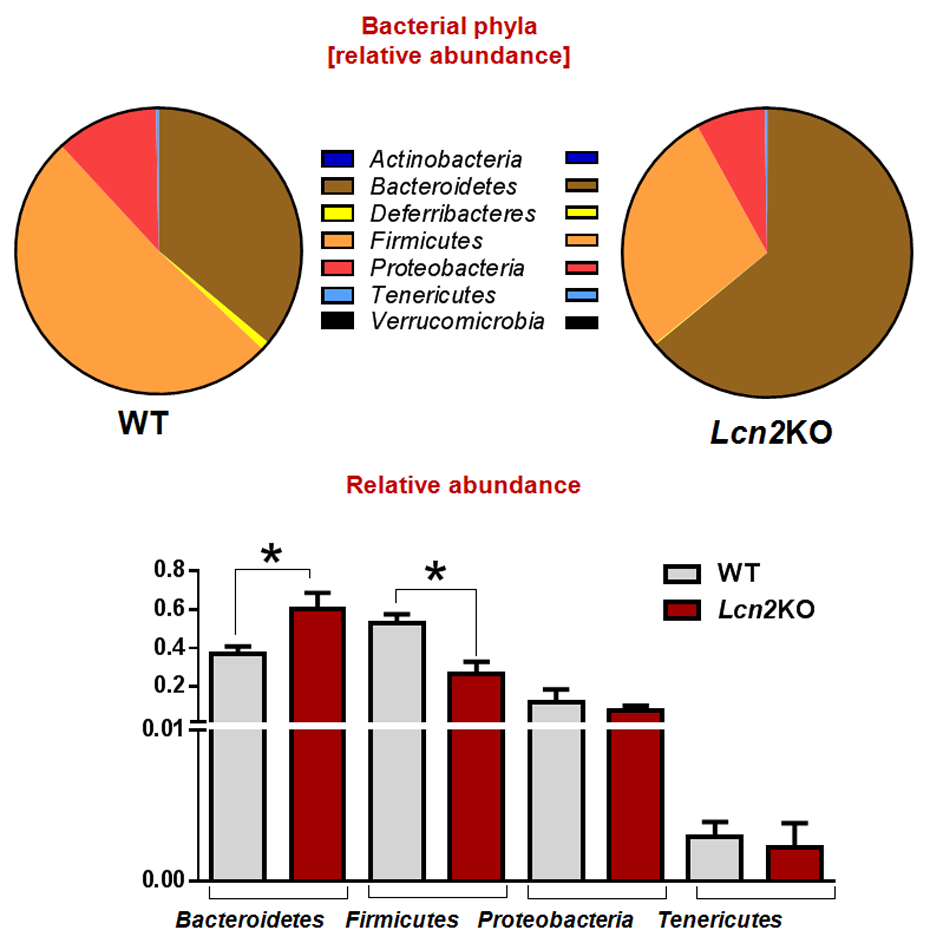
**

**A**

**B**

**Fig. S1: Lcn2 deficient mice display altered gut bacterial composition.** Gut bacterial composition was analyzed in eight weeks old male BALB/c *Lcn2*KO mice and their WT littermates using 16S rRNA gene pyrosequencing. **A-B**) Image displays the relative abundance of major bacterial phyla in BALB/c *Lcn2*KO mice and their WT littermates. The values are expressed as mean ± SEM (*p<0.05).
